# Binary effects of fluoxetine and zinc on the biomarker responses of the non-target model organism *Daphnia magna*

**DOI:** 10.1007/s11356-024-32846-5

**Published:** 2024-03-25

**Authors:** Gülüzar Atli, Yusuf Sevgiler

**Affiliations:** 1 Vocational School of İmamoğlu, Çukurova University, Adana, Turkey; 2 Biotechnology Research and Application Center, Çukurova University, Adana, Turkey; 3 Faculty of Science and Letters, Department of Biology, Adiyaman University, Adiyaman, Turkey

**Keywords:** ATPase, Integrated biomarker response index, Metals, Pharmaceuticals, Oxidative stress

## Abstract

The antidepressant effect of zinc on mammals has been documented in recent decades, and the concentration of the antidepressant fluoxetine (FLX) in aquatic environments has been rising constantly. The aim of the present study is to evaluate the combined toxicity of a serotonin reuptake inhibitor (FLX) and Zn^2+^ on a non-target aquatic model organism *Daphnia magna*. Animals were exposed to single and binary combinations of FLX (20.5 and 41 µg/L for subchronic and 41 and 82 µg/L for acute exposures) and Zn^2+^ (40 µg/L for subchronic and 80 µg/L for acute exposures). In vivo experiments were done for 7 days subchronic and 48 h acute exposure, while subcellular supernatants of whole Daphnia lysate (WDL) were directly treated with the same concentrations used in the acute experiments. Morphological characteristics, Ca^2+^-ATPase, antioxidant enzyme activities, and lipid peroxidation were examined. There was antioxidant system suppression and Ca^2+^-ATPase inhibition despite the diverse response patterns due to duration, concentration, and toxicant type. After acute exposure, biomarkers showed a diminishing trend compared to subchronic exposure. According to integrated biomarker response index (IBR) analysis, in vivo Zn^2+^ exposure was reasonably effective on the health of *D. magna*, whereas exposure of WDL to Zn^2+^ had a lesser impact. FLX toxicity increased in a concentration-dependent manner, reversed by the combined exposure. We concluded that potential pro-oxidative and adverse Ca^2+^-ATPase effects of FLX and Zn^2+^ in *D. magna* may also have harmful impact on ecosystem levels. Pharmaceutical exposure (FLX) should be considered along with their potential to interact with other toxicants in aquatic biota.

**Graphical Abstract:**

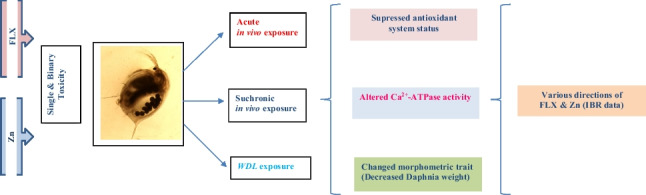

**Supplementary Information:**

The online version contains supplementary material available at 10.1007/s11356-024-32846-5.

## Introduction

*Daphnia magna* is an important component of aquatic biota which is a critical link between primary producers and higher-level consumers in freshwater ecosystems (Cox et al. 2018). This species is frequently found in these environments and is frequently employed as a model organism in toxicological research (OECD 2004); as a result, its possible reactions to a wide variety of pollutants are well-known (Campos et al. 2012b; Ding et al. 2017). Moreover, *D. magna* is the most studied model organism to determine the aquatic toxicity of pharmaceuticals (Bergmann et al. 2011). Hence, the rationale behind choosing this species for investigating the combined effects of aquatic toxicants, such as specific serotonin reuptake inhibitors (SSRIs), may be justified in the absence of an exhaustive exploration of detailed mechanistic pathophysiological pathways. Concurrently, certain physiological mechanistic details at the gene and/or protein level for this species have already been elucidated. For example, the presence of serotonin and functionally related proteins in daphnid species has been demonstrated by different studies (Ehrenström and Berglind 1988; McCoole et al. 2012). Therefore, any substance that modifies serotonin activity can subtly change these species’ regular endocrine and metabolic processes.

The daily dose of antidepressants taken per 1000 people across all OECD member nations showed a steady rise in usage (OECD Statistics 2021). One of the top 10 most given antidepressant medications is fluoxetine (FLX), an SSRI (Barakat et al. 2018). By binding to serotonin transporter proteins (SERT), FLX prevents the reuptake of serotonin at the presynaptic opening (Roman et al. 2003). FLX has been identified often in aquatic environments across the globe (Vasskog et al. 2008; Metcalfe et al. 2010; Mezzelani et al. 2018), in concentrations that can reach up to 0.6 µg/L (Hughes et al. 2013). In addition, higher FLX and its metabolite norFLX have been documented recently in wastewater treatment plant influents (3.5 and 10.4 µg/L, respectively) (Mole and Brooks 2019). FLX has accumulation potential in *D. magna* (Ding et al. 2017). It was underlined that FLX can easily enter the body with the nutrient flow and be taken up via the thin wall of the carapace during respiratory gas exchange in this species because of its thin exoskeleton and high surface area/volume ratio.

Numerous biological endpoints including growth and reproduction, oxidative stress response, and transcriptomic and metabolomic responses have been analyzed in studies investigating the effects of FLX in daphnid species (Flaherty and Dodson 2005; Campos et al. 2012a, 2016; Jordão et al. 2016; Ding et al. 2017; Stremmel et al. 2023). According to these studies, it is understood that FLX has a harmful potential on daphnid populations. After 7 days of exposure, superoxide dismutase (SOD) activity was reduced by 0.5 µg/L and increased by 5 µg/L FLX, although lipid peroxidation levels showed an opposite trend in *D. magna* (Ding et al. 2017). No alterations were observed in glutathione *S*-transferase (GST) activity or lipid peroxidation levels in *D. magna* following a 72-h exposure to 1 and 5 mg/L of FLX (Varano 2014). We previously demonstrated that FLX at environmental concentration (0.091 µg/L) has no prooxidant potential in *D. magna* after 21 days of chronic exposure (Över et al. 2020). However, glutathione peroxidase (GPX) activity and lipid peroxidation levels increased at 10 × and 100 × of this concentration after 4 and 21 days of exposure. Exposure to FLX at concentrations of 40 and 80 µg/L has been associated with various documented biological consequences (Campos et al. 2012a, 2016). According to Stremmel et al. (2023), FLX concentrations in aquatic environments will eventually reach lethal concentrations (up to 800 µg/L) in the worst-case scenario for future projections; therefore, we selected higher concentrations than current environmental levels for this study.

The antidepressant-like effect of Zn in mammals has been recognized for several decades. This element is a natural component of aquatic habitats. Human activity can cause the concentration of Zn to increase to dangerous levels (Trevisan et al. 2014). The possibility of coexistence of Zn with some pharmaceuticals like antidepressant FLX in aquatic environments is increasing with the increased usage in human treatments. Moreover, combined therapeutic treatment with Zn and antidepressant drugs like FLX in humans was proposed by many authors because of synergistic effects (Cunha et al. 2008; Refaey et al. 2015), while concentration-dependent antagonistic effects were presented (García-Colunga et al. 2005) as explained below. Therefore, the assessment of combined toxicity of these substances may be indicative of aquatic ecosystem health, as Zn has the potential to amplify the effects of low environmental concentrations of antidepressants.

Zinc, an essential micronutrient, plays multifaceted roles in physiological pathways. (Muyssen and Janssen 2002; Oteiza 2012). However, its excess can induce neurotoxicity in various animal groups such as crustaceans and mammals (Szewczyk 2013; Kukavica et al. 2023), while deficiency in mammals elevates the risk of neurological disorders like depression (Szewczyk 2013). In mammals, Zn can affect a range of receptors or transporters at pre- and post-synaptic sides (Doboszewska et al. 2017). For example, it exhibits a concentration-dependent biphasic effect on agonist binding to the serotonin receptor 5-HT_1a_ (Satała et al. 2016). The antidepressant-like effect of zinc is impeded by serotonin synthesis inhibition or specific receptor antagonism (Szewczyk et al. 2009). Structural disparities in serotonin receptors between vertebrates and arthropods (and other invertebrates) suggest functional variations (Tierney 2001). *D. pulex* have been found to express 5-HT_1a_ receptors, which are structural and functional similarity with Drosophila 5-HT_1a_ receptors (McCoole et al. 2012). It has been suggested that Zn may interact with these receptors to influence the physiological processes related to serotonin in these species.

The stress brought about by toxicant exposure triggers many biological reaction mechanisms and simultaneous analysis of numerous biomarkers can provide valuable insights into the stress and toxicity mechanism of a particular toxicant, offering a comprehensive understanding of their effects on biological systems (Van der Oost et al. 2003). These biomarkers play a crucial role in various stages of the risk assessment process, including effect, exposure, and hazard assessment, as well as risk characterization or classification, and monitoring the environmental quality of aquatic ecosystems (Iturburu et al. 2018). Determining the susceptibility of an organism to a specific substance through biomarkers poses challenges without a statistical approach to evaluate the global trend of toxicity. According to Devin et al. (2014), the integrated biomarker response index (IBR) approach yields a graphical synthesis of the various responses and a numerical value that simultaneously incorporates all the biomarker responses. The IBR method is an effective tool for visualization of the pollutant impact and simplifying the interpretation of relationships between multiple biomarkers of different pathways and exposure levels (Devin et al. 2014), especially when biomarkers within the same metabolic pathway show no correlation across different exposure scenarios (Potet 2017). Additionally, it aids in evaluating the sensitivity of an organism, tissue, or cell to a toxicant or its concentrations by considering the responses of various biomarkers (Kim et al. 2014). Oxidative stress-related processes garner significant attention due to their sensitivity and prevalence in numerous pathophysiological situations (Van der Oost et al. 2003). The most studied parameters to comprehend the prooxidative nature of toxicants are SOD, catalase (CAT), antioxidant tripeptide glutathione (GSH), and its related enzymes GPX and GST, as well as lipid peroxidation levels (measured as thiobarbituric acid reactive substances [TBARS]). In *D. magna*, ATPase enzymes were successfully used as a sensitive biomarker of metal and pesticide exposures in earlier studies because they are crucial for ionic equilibrium (Mahmut et al. 2022; Sevgiler and Atli 2022). For example, there is an active Ca^2+^ cycle in daphnid species because of loss of 90% of their calcium with their carapace during moulting period, and Ca^2+^-ATPase is important for active transport of calcium into the body (Cox et al. 2018).

Fluoxetine and Zn co-administration can affect the antioxidant response in mammals. Separate Zn treatment had no effect on the expression of the genes involved in the antioxidant system, such as nuclear factor-erythroid 2-related factor 2 (Nrf-2) and heme oxygenase-1 (HO-1). However, FLX exhibited an antioxidant-response inducing effect, leading to enhanced expression of these genes. The metallothionein gene’s expression rose in the presence of these substances, and the greatest rise was found in the FLX and Zn combined treatment group (Omar and Tash 2017). These results increase the possibility that FLX and Zn may also have an impact on the antioxidant system in non-target species like *D. magna*. Using biomarkers of stress, we attempted to explain the combined toxicity of two distinct chemicals, which might be connected in their therapeutic/toxic mechanism, in a model non-target aquatic organism. To identify potential toxicity, morphometric characteristics, Ca^2+^-ATPase, and oxidative stress-related biomarkers were analyzed; and the IBR approach was used to identify the global trend in biological responses to these compounds and their combinations in *D. magna*.

## Materials and methods

### Animal husbandry

Animals were supplied by the Carolina Biological Supply Company, located in Burlington, North Carolina, and were cultured in our facility for almost 7 years prior to the experiments. Seventy-two *D. magna* samples were divided into two cassettes, each containing 3000 mL of hard water per the American Society for Testing and Materials International's guidelines (ASTM 2002). These animals were less than 24-h old and the third offspring of their mothers, who were the fifth-generation nestlings of one mother. The water’s physicochemical properties were pH 7.8–8.0, hardness 160–180 mg/L as CaCO_3_, and alkalinity 110–120 mg/L as CaCO_3_ (ASTM 2002). The culture was maintained under 12.15 µmol m^−2^ s^−1^ light with 16 h: 8 h light: dark photoperiod at 21 °C (Cleresci et al. 1999). The mixture of trout chow, yeast, dried alfalfa (Cleresci et al. 1999), and Roti-Rich™ were used for feeding. Animals were fed every other day at a rate of 1.5 mL/L trout chow-yeast-alfalfa mixture and 200 µL/L Roti-Rich™. After a week, the water was replaced with brand-new ASTM hard water. The first generation was removed and not used in toxicity tests. Prior to the toxicity tests, a typical 24-h EC_50_ sensitivity test was carried out using the second offspring and K_2_Cr_2_O_7_. The calculated EC_50_ fell within the interval recommended by the OECD (Organization for Economic Co-operation and Development, (OECD 2004) as 1.108 mg/L (C.I. 1.066–1.140 mg/L). The third offspring were used in subchronic toxicity experiments. For the acute and whole Daphnia lysate (WDL) treatment experiments, 860, less than 24-h old neonates of the fourth offspring were divided equally into three different cassettes containing 12 L ASTM hard water. The cassettes were gently aerated with filtered ambient air. As noted, the animals were fed. After 1 week, the water was replaced with brand-new ASTM hard water. When these animals were 10 days old, acute toxicity experiments were started or they collected for WDL treatment when they were 14 days old.

### Toxicity experiments

Less than 24-h or 10 days old, third or fourth offspring animals were divided into six groups for semi-static subchronic (i.e., 7-d) or acute (i.e., 48-h) toxicity experiments, respectively. For each group, five glass beakers with 800 mL of ASTM hard water served as replicates. There were 21 or 20 animals placed into each beaker for subchronic or acute tests, respectively. Stock FLX solution was prepared with fluoxetine hydrochloride, which was a pharmaceutical secondary standard grade (CAS No: 56296–78-7, Supelco). Zn solution was prepared with ZnCl_2_ (CAS No: 7646–85-7, Sigma-Aldrich). The 48-h EC_50_ values of FLX and Zn for *D. magna* were 820 µg/L and 800 µg/L, respectively (Diamantino et al. 2001; Brooks et al. 2003). These values were considered as 1 toxic unit (TU). The sublethal 0.025 and 0.05 TU values of FLX were selected for subchronic (corresponding to 20.5 and 41 µg/L, respectively), while 0.05 and 0.1 TU values for acute and WDL experiments (corresponding to 41 and 82 µg/L, respectively). Zn concentrations were 0.05 and 0.1 TU values corresponding to 40 µg/L and 80 µg/L for subchronic or acute and WDL toxicity experiments, respectively.

The water used in the subchronic experiments was entirely replaced with fresh medium every other day, and 1.5 mL/L of a trout chow-yeast-alfalfa mixture and two drops of Roti-Rich™ were added for animal feeding. Only at the beginning of acute studies where experimental animals fed. The experimental water was totally replaced after 24-h and animals were not fed during this time. Throughout the experimentation, no animal perished.

The physicochemical properties of the experimental water for subchronic experiments were as follows: pH 7.97 ± 0.02, the temperature 21.58 ± 0.78 °C, dissolved oxygen 9.86 ± 0.09 mg/L, and conductivity 544.00 ± 4.55 µS/cm. For acute tests, the corresponding values were pH 7.99 ± 0.02, the temperature 21.53 ± 0.21 °C, dissolved oxygen 9.94 ± 0.16 mg/L, and conductivity 546.67 ± 9.87 µS/cm. Twenty animals from each beaker were collected at the conclusion of the exposure durations, pooled as one replication; the extra water was blotted out with tissue paper, the animals were weighed, and they were kept at – 80 °C until biochemical analyses. The pooled animals underwent homogenization (1:10 w/v) for 2 min on ice using a stainless steel homogenizer in a chilled homogenisation buffer (250 mM sucrose, 20 mM Tris, 1 mM EDTA, pH 7.8). Homogenates were centrifuged at 13,000 × *g* for 20 min at 4 °C. In the supernatant, total protein concentrations and enzyme activities with TBARS and GSH levels were assessed spectrophotometrically.

The direct effects of FLX and Zn^2+^ on the selected biomarkers by reducing the organismal and/or cellular borders, 13,000 × *g* supernatants of *D. magna* were divided into the groups mentioned in acute experiments. After this point, this experimental setting was named as “WDL.” A total of 36 14-day-old animals per group were aggregated in microtubes, subjected to homogenization, and centrifuged to assess the WDL effect. Six microtubes were prepared for this purpose. To compare the outcomes of acute in vivo and WDL effects, acute exposure concentrations were employed. Each toxicant was promptly mixed with the acquired supernatant, and a 30-min incubation period was allowed at room temperature. For a minimum of triplicate biochemical examination, the processed supernatant was utilized.

### Morphometric characteristics analysis

After subchronic experiments, one animal from each beaker was randomly selected and collected in tubes containing 50% ethanol and kept at 4 °C until morphometric analysis. Concave microscope slides containing lactic acid solutions were used. Animals were captured on camera using an Olympus BX53 binocular microscope that has a Canon EOS1200D camera attached. The caudal spine was excluded from this measurement, which assessed the length of the carapace between the anterior head and the base of the spine (Zhu et al. 2010). This morphometric analysis was conducted as an additional toxicity parameter. Maximum carapace width was measured laterally from the entire width of carapace ends. Micam v3.0 imaging software (http://www.science4all.nl/?Microscopy_and_Photography) was used for scaling. An analytical balance (HR250AZ, AND Company Ltd., Tokyo, Japan) was used to measure the combined weight of the pooled animals that were gathered for biochemical examination. The average weight of one animal was subsequently expressed as mg of wet weight.

### Biochemical analysis

Ca^2+^-ATPase activity was measured in an incubation medium (pH 7.7) comprising 40 mM Tris–HCl, 4 mM MgCl_2_, 1 mM CaCl_2_, and 1 mM ethylene glycol‐bis (2‐aminoethyl ether)‐,*N*,*N′*,*N′*‐tetra acetic acid (EGTA). Then, 8 μL of supernatant was added to incubation medium, reaching a total volume of 250 µL, and preincubated for 5 min at 37 °C. The reaction was initiated by adding 25 μL Na_2_ATP (3 mM) and incubating for 30 min. To stop the reaction, 125 μL of ice‐cold distilled water was added. Inorganic phosphate (Pi) was measured as described by Atkinson et al. (1973). Appropriate blanks were included with each assay to correct the non-enzymatic hydrolysis of ATP. KH_2_PO_4_ (25–250 μM) was used as the Pi standard and the spectrophotometric analysis was performed at 390 nm. The Ca^2+^-ATPase activity was quantified as the absorbance difference between the presence and absence of CaCl_2_, expressed as µmol Pi/mg protein/h.

SOD activity was measured by the indirect method, involving the inhibition of cytochrome *c* reduction at 550 nm for 1 min (McCord and Fridovich 1969). The reaction buffer, in a final volume of 1 mL, comprised 50 mM potassium phosphate buffer (pH 7.0), 0.1 mM EDTA, 10 µM cytochrome *c*, 0.05 mM hypoxanthine, and 10 µL of the supernatant. The reaction was started by adding 1.88 mU/mL xanthine oxidase (XOD), with the XOD blank was used to adjust the XOD concentration. SOD activity was expressed as unit/mg protein, defined as the amount of enzyme that causes 50% inhibition of cytochrome *c* reduction.

CAT activity was measured according to the method of Lartillot et al. (1988). Twenty μL of supernatant, containing 0.2 mg protein/mL, were mixed with 2.5 mL of 25 mM H_2_O_2_ in a 75 mM phosphate buffer at pH 7.0. The CAT activity was calculated as μmol H_2_O_2_ decomposed/mg protein/min, using a specific absorption coefficient of 0.0392 cm^2^ μmol^−1^ H_2_O_2_, at 240 nm.

GPX activity was measured in a 1 mL reaction buffer consisting of 100 mM potassium phosphate buffer (pH 7.0), 2 mM GSH, 0.12 mM NADPH, 2 U glutathione reductase (GR), 10 µL supernatant, and 3 mM cumene hydroperoxide (Livingstone et al. 1992). The activity was expressed as µmol/mg protein/min estimating the decrease of NADPH per minute at 340 nm, with a specific absorption coefficient is 6.22 M^−1^ cm^−1^ NADPH at 340 nm.

GST activity was evaluated by an increase of absorbance per minute at 340 nm resulting from the conjugation of GSH and 1-chloro-2,4-dinitrobenzene (CDNB) (Habig et al. 1974). The specific absorption coefficient of the conjugate *S*-(2,4-dinitrophenyl)-glutathione is 9.6 mM^−1^ cm^−1^ at 340 nm. In a 1-mL reaction buffer containing 100 mM potassium phosphate buffer (pH 7.5), 1 mM GSH, 1 mM CDNB, and 10 µL supernatant, GST activity was expressed as µmol/mg protein/min.

GSH levels were analyzed by measuring the absorbance increase at 412 nm per minute and expressed as micromoles of GSH equivalents per milligrams of protein (Griffith 1980). A standard curve was prepared with GSH, and the reaction buffer in a final volume of 1 mL contained 100 mM sodium phosphate buffer (pH 7.5), 4.2 mM NADPH, 0.8 mM DTNB, 75 U/mL GR, and 25 µL supernatant.

Lipid peroxidation levels was assessed using the TBARS assay, which measures the lipid peroxidation products that react with thiobarbituric acid (TBA). The TBARS analysis was performed based on the incubation of supernatants and TBA in aerobic conditions at the temperature of 100 °C. TBARS concentrations were determined by measuring the formation of pink-coloured complex at 532 nm (Wills 1966). The calculations were based on an external standard curve generated using 1,1′,3,3′-tetramethoxypropane, and the values were expressed in nanomoles/mg protein.

Total protein content was measured spectrophotometrically at 750 nm using the Lowry method (Lowry et al. 1951), with bovine serum albumin serving as an external standard. The total protein content was expressed as mg per mL supernatant. All assays were conducted in duplicate.

### Integrated biomarker response index calculations

The integrated biomarker response index (IBR), as described by Devin et al. (2014), was calculated to combine all results from various biomarkers and comprehend global/general reactions. We used the code presented on the website (https://liec-univ-lorraine.shinyapps.io/calibri/) by Devin et al. (2023). Briefly, the general mean (*m*) and standard deviation (*s*) for each biomarker were calculated across all data. Subsequently, a standardization was employed for each circumstance to yield *Y*, where *Y* = (*X* − *m*)/*s*, where *X* is the mean value for the biomarker at a specific group. The *Z* value was then determined using *Z* =  − *Y* or *Z* = *Y*, signifying inhibition or stimulation, respectively, in the case of a biological effect. The *S* value was calculated using the formula *S* = *Z* +|Min|, where Min represents the smallest value observed across all exposure groups for each biomarker in a specific exposure time. The *S*_*i*_ values, representing the standardized values of each biomarker, were plotted on a radar diagram. The total area depicted on the radar diagram is used to calculate the IBR. When seven or eight biomarkers are considered in a *k*-biomarker study, the area of the triangle formed by two subsequent biomarkers is defined as follows:

$${A}_{i} = {S}_{i}\times {S}_{i+1} \times \mathrm{ sin}(2\uppi /k)/2$$

The IBR value is then determined as follows:

$${\text{IBR}}={\sum }_{i=1}^{k}Ai$$

Due to the neighborhood effect, all conceivable circular permutations of *k* biomarkers were taken into consideration when calculating IBR values for subchronic, acute, or WDL exposure regimes. For all exposure regimes, the same set of parameters were used in the IBR calculations except for the animal weight in subchronic duration. This adjustment was necessitated by the 8-biomarker limitation in the code.

### Statistical analysis

Statistical analysis was performed in IBM SPSS Statistics 23 statistical software. The Kolmogorov–Smirnov normality test was applied before further analysis. One-way ANOVA was applied for normally distributed data before Levene’s homogeneity of variance test. All the data were categorised as homogeneous subsets, and then Duncan’s post hoc multiple range tests were used. Mann–Whitney *U* test was applied after the Kruskal–Wallis analysis for non-normal distributed data. All the results were given as mean ± standard error (*N* = 5). Spearman *rho* correlation was also applied to compare the studied parameters’ correlations (*P* < 0.05). Paired two-sample *t*-test was used to compare the IBR values in a specific exposure regime.

## Results

Table 1 shows the subchronic effect of FLX, Zn, or their mixtures on the morphometric traits of *D. magna*. The maximum carapace width and length remained unaffected. Subchronic Zn_0.05TU_ exposure decreased the caudal spine length in comparison to the control and FLX_0.025TU_ + Zn_0.05TU_ by 15% and 14%, respectively (*P* < 0.05). All exposed daphnids were impacted by Zn exposure in comparison to the control group, leading to weight decreases of 6.5%, 11% (*P* < 0.05), and 14% (*P* < 0.05) in the groups exposed to Zn_0.05TU_, FLX_0.025TU_ + Zn_0.05TU_, and FLX_0.05TU_ + Zn_0.05TU_, respectively.

**Table 1:** Morphometric characteristics of *D. magna* upon single and binary subchronic exposure to FLX and Zn^2+^

	Carapace length (µm)	% of cont	Max. carapace width (µm)	% of cont	Caudal spine length (µm)	% of cont	Weight (mg)	% of cont
Control	280.20 ± 4.38		192.20 ± 2.40		77.40 ± 0.93^**a**^		2.45 ± 0.02^**ab**^	
FLX_0.025TU_	275.40 ± 2.21	− 1.7	199.00 ± 5.03	3.5	70.00 ± 3.33^**ab**^	− 9.6	2.64 ± 0.06^**a**^	7.8
FLX_0.05TU_	282.20 ± 4.42	0.7	193.80 ± 3.28	0.8	75.20 ± 3.44^**a**^	− 2.8	2.66 ± 0.06^**a**^	8.6
Zn_0.05TU_	278.80 ± 3.89	− 0.5	193.00 ± 4.01	0.4	65.80 ± 2.92^**b**^	− **15.0**	2.29 ± 0.11^**bc**^	− 6.5
FLX_0.025TU_ + Zn_0.05TU_	278.20 ± 4.83	− 0.7	192.80 ± 6.40	0.3	76.60 ± 0.93^**a**^	− 1.0	2.17 ± 0.07^**c**^	− **11.4**
FLX_0.05TU_ + Zn_0.05TU_	272.60 ± 5.32	− 2.7	182.00 ± 3.72	− 5.3	71.00 ± 3.11^**ab**^	− 8.3	2.10 ± 0.06^**c**^	− **14.3**

Data were given as mean ± standard error of mean. There are statistical differences between groups in the same column that do not share the same letters (*N* = 5, *P* < 0.05). Statistically significant relative changes compared to control were given as percentages written in bold (*P* < 0.05)

Subchronic FLX_0.025TU_ exposure caused a 36% decrease in Ca^2+^-ATPase activity compared to the control (*P* < 0.05). However, there was an increased activity of 30–63% upon FLX_0.05TU_ + Zn_0.05TU_ exposure compared to the other groups (*P* < 0.05), excluding the control (*P* > 0.05). Ca^2+^-ATPase activity enhanced in the range of 39–161% following acute FLX_0.05TU_ exposure compared to the other groups (*P* < 0.05), while Zn significantly declined the activity to 44–62% in comparison with all groups apart from the FLX_0.1TU_ group (*P* < 0.05). WDL exposure significantly reduced the Ca^2+^-ATPase activity in the percent range of 42–72 in all groups in contrast to the control (*P* < 0.05). On the other hand, Ca^2+^-ATPase activity exhibited a declining value after Zn (84–112%) and Zn + FLX exposures (51–112%) compared to the single FLX effect (*P* < 0.05) (Table 2).

**Table 2:** Ca^2+^-ATPase-specific activities (µmol Pi/mg protein/h) of *D. magna* upon single and binary exposures to FLX and Zn^2+^

	Subchronic	% of cont		Acute	% of cont	WDL	% of cont
Control	6.73 ± 0.23^**ac**^		**Control**	11.62 ± 0.58^**a**^		5.58 ± 0.35^**a**^	
FLX_0.025TU_	4.33 ± 0.70^**b**^	− **35.6**	**FLX**_**0.05TU**_	16.13 ± 1.56^**b**^	**38.8**	2.85 ± 0.24^**b**^	− **48.9**
FLX_0.05TU_	5.18 ± 0.64^**ab**^	− 23.0	**FLX**_**0.1TU**_	8.00 ± 1.00^**ac**^	− 31.1	3.26 ± 0.23^**b**^	− **41.6**
Zn_0.05TU_	5.30 ± 0.45^**ab**^	− 21.3	**Zn**_**0.1TU**_	6.18 ± 0.71^**c**^	− **46.8**	1.77 ± 0.27^**c**^	− **68.3**
FLX_0.025TU_ + Zn_0.05TU_	5.43 ± 0.37^**ab**^	− 19.3	**FLX**_**0.05TU**_** + Zn**_**0.1TU**_	10.94 ± 2.13^**a**^	− 5.9	1.54 ± 0.16^**c**^	− **72.4**
FLX_0.05TU_ + Zn_0.05TU_	7.04 ± 0.32^c*^	4.6	**FLX**_**0.1TU**_** + Zn**_**0.1TU**_	10.98 ± 1.76^**a**^	− 5.5	1.89 ± 0.25^**c**^	− **66.1**

Data were given as mean ± standard error of mean. There are statistical differences between groups in the same column that do not share the same letters. (*N* = 4–5, *N = 3; *P* < 0.05). Statistically significant relative changes compared to control were given as percentages written in bold (*P* < 0.05)

SOD activity increased by 24% in the FLX_0.05TU_ group and by 27–28% in the FLX_0.05TU_ + Zn_0.05TU_ group, contrasting with the FLX_0.025TU_ and Zn_0.05TU_ groups, respectively after subchronic exposure (*P* < 0.05). However, its activity decreased in Zn_0.1TU_ and FLX_0.05TU_ + Zn_0.1TU_ groups when compared to the control, with a range of 40–43% (*P* < 0.05), and compared to single FLX groups, with a range of 48–54% (*P* < 0.05), upon acute exposure. WDL exposure inhibited SOD activity in all groups ranging from 17 to 34% compared to the control (*P* < 0.05). Notably, SOD activity in the FLX_0.05TU_ group was higher by 17% and 26% than in the Zn_0.1TU_ and FLX_0.05TU_ + Zn_0.1TU_ groups, respectively (*P* < 0.05) (Table 3, 4, and 5).

**Table 3:** Subchronic effects of FLX, Zn^2+^, and their combinations on antioxidant enzyme specific activities, GSH, TBARS, and total protein levels

	SOD	CAT	GPX	GST	GSH	TBARS	Protein
Control	7.29 ± 0.52^**ab**^	19.48 ± 1.14^**a**^	0.011 ± 0.001^**a**^	0.025 ± 0.002^**ab**^	1.35 ± 0.26^**a**^	0.49 ± 0.03^**a**^	9.56 ± 0.50
FLX_0.025TU_	6.59 ± 0.49^**a**^	12.05 ± 1.24^**b**^	0.006 ± 0.001^**b**^	0.026 ± 0.001^**ab**^	2.01 ± 0.36^**b**^	0.54 ± 0.04^**ab**^	9.81 ± 0.30
FLX_0.05TU_	8.15 ± 0.36^**b**^	19.08 ± 1.40^**a**^	0.007 ± 0.001^**b**^	0.027 ± 0.001^**a**^	0.57 ± 0.09^**c**^	0.55 ± 0.05^**ab**^	9.62 ± 0.41
Zn_0.05TU_	6.55 ± 0.36^**a**^	19.42 ± 2.44^**a**^	0.011 ± 0.001^**a**^	0.020 ± 0.001^**c**^	0.79 ± 0.10^**c**^	0.51 ± 0.04^**a**^	8.67 ± 0.58
FLX_0.025TU_ + Zn_0.05TU_	7.26 ± 0.61^**ab**^	13.24 ± 3.17^**ab**^	0.011 ± 0.002^**a**^	0.026 ± 0.002^**ab**^	0.61 ± 0.05^**c**^	0.45 ± 0.02^**a**^	9.24 ± 0.43
FLX_0.05TU_ + Zn_0.05TU_	8.40 ± 0.30^**b**^	17.85 ± 1.67^**ab**^	0.009 ± 0.000^**ab**^	0.022 ± 0.001^**bc**^	0.00 ± 0.00^**d**^	0.63 ± 0.03^**b**^	9.08 ± 0.45
	**SOD**	**CAT**	**GPX**	**GST**	**GSH**	**TBARS**	**Protein**
FLX_0.025TU_	− 9.6	− **38.1**	− **45.5**	4	**48.9**	10.2	2.6
FLX_0.05TU_	11.8	− 2.1	− **36.4**	8	− **57.8**	12.3	0.6
Zn_0.05TU_	− 10.2	− 0.3	0	− **20**	− **41.5**	4.1	− 9.3
FLX_0.025TU_ + Zn_0.05TU_	− 0.4	− 32.0	0	4	− **54.8**	− 8.2	− 3.4
FLX_0.05TU_ + Zn_0.05TU_	15.2	− 8.4	− 18.2	− 12	− **100.0**	**28.6**	− 5.0

Data were given as mean ± standard error of mean. There are statistical differences between groups in the same column that do not share the same letters. (*N* = 4–5; *P* < 0.05). Statistically significant relative changes compared to control were given as percentages written in bold (*P* < 0.05). SOD (unit/mg protein), CAT (μmol H_2_O_2_ decomposed/mg protein/min), GPX and GST (µmol/mg protein/min), GSH (μmol GSH equivalents/mg protein), TBARS (nmol/mg protein), and protein (mg/mL)

**Table 4:** Acute effects of FLX, Zn^2+^, and their combinations on antioxidant enzyme specific activities, GSH, TBARS, and total protein levels

	SOD	CAT	GPX	GST	GSH	TBARS	Protein
Control	8.64 ± 0.43^**a**^	15.51 ± 0.91^**ab**^	0.023 ± 0.003^**a**^	0.014 ± 0.001	0.37 ± 0.05^**a**^	0.81 ± 0.04^**ab**^	4.74 ± 0.22^**a**^
FLX_0.05TU_	10.00 ± 1.10^**a**^	18.14 ± 1.51^**b**^	0.013 ± 0.001^**b**^	0.017 ± 0.003	0.22 ± 0.06^b*^	0.74 ± 0.03^**a**^	5.41 ± 0.23^**b**^
FLX_0.1TU_	10.70 ± 0.86^**a**^	12.84 ± 0.83^**ac**^	0.013 ± 0.000^b*^	0.014 ± 0.005	0.00 ± 0.00^**c**^	0.69 ± 0.04^**a**^	5.54 ± 0.21^**bc**^
Zn_0.1TU_	4.94 ± 1.76^**b**^	13.96 ± 0.99^**ac**^	0.013 ± 0.001^b*^	0.015 ± 0.002	0.00 ± 0.00^**c**^	0.69 ± 0.03^**a**^	6.00 ± 0.07^**bc**^
FLX_0.05TU_ + Zn_0.1TU_	5.17 ± 1.25^**b**^	11.49 ± 1.16^**c**^	0.000 ± 0.000^**c**^	0.015 ± 0.002	0.00 ± 0.00^**c**^	0.71 ± 0.03^**a**^	5.95 ± 0.18^**bc**^
FLX_0.1TU_ + Zn_0.1TU_	8.00 ± 0.70^**ab**^	13.10 ± 1.15^**ac**^	0.008 ± 0.002^**b**^	0.015 ± 0.004	0.00 ± 0.00^**c**^	0.95 ± 0.12^**b**^	6.21 ± 0.31^**c**^
	**SOD**	**CAT**	**GPX**	**GST**	**GSH**	**TBARS**	**Protein**
FLX_0.05TU_	15.7	17.0	− **43.5**	21.4	− **40.5**	− 8.6	**14.1**
FLX_0.1TU_	23.8	− 17.2	− **43.5**	0.0	− **100.0**	− 14.8	**16.9**
Zn_0.1TU_	− **42.8**	− 10.0	− **43.5**	7.1	− **100.0**	− 14.8	**26.6**
FLX_0.05TU_ + Zn_0.1TU_	− **40.2**	− **25.9**	− **100.0**	7.1	− **100.0**	− 12.4	**25.5**
FLX_0.1TU_ + Zn_0.1TU_	− 7.4	− 15.5	− **65.2**	7.1	− **100.0**	17.3	**31.0**

Data were given as mean ± standard error of mean. Different letters demonstrate the statistical differences among exposure groups (*N* = 4–5, *N = 3; *P* < 0.05). Statistically significant relative changes compared to control were given as percentages written in bold (*P* < 0.05). SOD (unit/mg protein), CAT (μmol H_2_O_2_ decomposed/mg protein/min), GPX and GST (µmol/mg protein/min), GSH (μmol GSH equivalents/mg protein), TBARS (nmol/mg protein), and protein (mg/mL)

**Table 5:** WDL effects of FLX, Zn^2+^, and their combinations on antioxidant enzyme specific activities, and GSH and TBARS levels

	SOD	% of cont	CAT	% of cont	GPX	% of cont	GST	% of cont	GSH	% of cont	TBARS	% of cont
Control	6.53 ± 0.31^**a**^		14.97 ± 0.57^**a**^		0.004 ± 0.001^**a**^		0.025 ± 0.002		0.85 ± 0.22^**a**^		0.11 ± 0.02^a*^	
FLX_0.05TU_	5.44 ± 0.15^**b**^	− **16.7**	12.12 ± 0.68^**b**^	− **19.0**	0.005 ± 0.001^**a**^	25	0.023 ± 0.001	− 8	0.72 ± 0.04^a*^	− 15.3	0.12 ± 0.01^ab*^	9.1
FLX_0.1TU_	4.74 ± 0.22^**bc**^	− **27.4**	12.85 ± 0.30^**bc**^	− **14.2**	0.007 ± 0.001^**a**^	75	0.022 ± 0.001	− 12	1.95 ± 0.18^**b**^	**129.4**	0.15 ± 0.01^**bc**^	**36.4**
Zn_0.1TU_	4.31 ± 0.14^**c**^	− **34.0**	12.17 ± 0.58^**b**^	− **18.7**	0.000 ± 0.000^**b**^	− **100**	0.021 ± 0.001	− 16	0.00 ± 0.00^**c**^	− **100.0**	0.14 ± 0.01^**bc**^	**27.3**
FLX_0.05TU_ + Zn_0.1TU_	4.67 ± 0.21^**c**^	− **28.5**	14.58 ± 0.09^**ac**^	− 2.6	0.000 ± 0.000^**b**^	− **100**	0.021 ± 0.002	− 16	0.04 ± 0.02^**c**^	− **95.3**	0.16 ± 0.01^**c**^	**45.5**
FLX_0.1TU_ + Zn_0.1TU_	4.93 ± 0.33^**bc**^	− **24.5**	11.83 ± 1.01^**b**^	− **21.0**	0.000 ± 0.000^**b**^	− **100**	0.021 ± 0.002	− 16	0.15 ± 0.09^**c**^	− **82.4**	0.15 ± 0.01^**bc**^	**36.4**

Data were given as mean ± standard error of mean. Different letters demonstrate the statistical differences among exposure groups (*N* = 4–5, *N = 3; *P* < 0.05). Statistically significant relative changes compared to control were given as percentages written in bold (*P* < 0.05). SOD (unit/mg protein), CAT (μmol H_2_O_2_ decomposed/mg protein/min), GPX and GST (µmol/mg protein/min), GSH (μmol GSH equivalents/mg protein), and TBARS (nmol/mg protein)

Subchronic FLX_0.025TU_ exposure decreased the CAT activity in contrast to the control, FLX_0.05TU_, and Zn_0.05TU_ groups in a percent range of 37–38% (*P* < 0.05). CAT activity increased upon acute FLX_0.05TU_ exposure in a range of 30–41% when compared to all groups (*P* < 0.05), except for the control (*P* > 0.05). However, the FLX_0.05TU_ + Zn_0.1TU_ group significantly decreased to 26% and 37% when compared to both the control and FLX_0.05TU_ groups, respectively (*P* < 0.05). WDL exposure also caused a decline in CAT activity of FLX_0.05TU_ (19%), FLX_0.1TU_ (14%), Zn_0.1TU_ (19%), and FLX_0.1TU_ + Zn_0.1TU_ (21%) groups when compared to the control (*P* < 0.05). On the other hand, FLX_0.05TU_ + Zn_0.1TU_ exposure led to a higher CAT activity in order of 20%, 20%, and 23% than the groups of FLX_0.05TU_, Zn_0.1TU_, and FLX_0.1TU_ + Zn_0.1TU_ (*P* < 0.05) (Table 3, 4, and 5).

In single FLX-exposed groups, GPX activity reduced by 33–47% when compared to the control, Zn_0.05TU_, and FLX_0.025TU_ + Zn_0.05TU_ groups after subchronic exposure (*P* < 0.05). GPX activity was significantly decreased in all exposed groups, ranging from 44 to 100%, in contrast to the control (*P* < 0.05) while acute FLX_0.05TU_ + Zn_0.1TU_ exposure completely inhibited it (*P* < 0.05). GPX activity remained unaffected in single FLX groups (*P* > 0.05), while its activity was totally inhibited (100%) in single Zn and combined groups when compared to the control after WDL exposure (*P* < 0.05) (Table 3, 4, and 5).

Subchronic Zn_0.05TU_ exposure caused a significant decline to 19–26% in GST activity in contrast to all exposed groups (*P* < 0.05) except the FLX_0.05TU_ + Zn_0.05TU_ group (*P* > 0.05). On the other hand, GST activity was higher at 36% and 22% in the FLX_0.05TU_ group compared to both Zn_0.05TU_ and FLX_0.05TU_ + Zn_0.05TU_ groups, respectively (*P* < 0.05) (Table 3). However, GST activity was not affected by both acute and WDL exposures (*P* > 0.05) (Table 4 and 5).

A decrease was observed in GSH level in subchronic exposure (*P* < 0.05) as well excluding the FLX_0.025TU_ group which was higher than all groups at a 49–100% range (*P* < 0.05). Similarly, following acute exposure, GSH levels declined to 41–100% across all groups in contrast to the control (*P* < 0.05). Additionally, FLX_0.05TU_ exposed group had higher GSH level than the other exposure groups (*P* < 0.05). WDL exposure also caused a GSH decrement (82–100%) in groups following single Zn_0.1TU_ and Zn + FLX exposures (*P* < 0.05), whereas its level increased after FLX_0.1TU_ exposure when compared to others (*P* < 0.05) (Table 3, 4, and 5).

Subchronic FLX_0.05TU_ + Zn_0.05TU_ exposure increased TBARS levels in comparison with the control (29%), Zn_0.05TU_ (23%), and FLX_0.025TU_ + Zn_0.05TU_ (40%) groups (*P* < 0.05) (Table 3). The same pattern was observed in acute exposure to FLX_0.1TU_ + Zn_0.1TU_ in the range of 28–38% after when compared to the other groups (*P* < 0.05) except for the control (*P* > 0.05) (Table 4). Elevated levels of TBARS (27–46%) were also observed in all exposed groups (*P* < 0.05) except the FLX_0.05TU_ group in contrast to the control (*P* > 0.05) after WDL exposure (Table 5). Among the groups, FLX_0.05TU_ + Zn_0.1TU_ exposure caused the highest TBARS levels.

Subchronic exposure caused any significant alteration in protein levels (*P* > 0.05) (Table 3). However, they were significantly stimulated (14–31%) by all exposed groups following acute exposure when compared to the control (*P* < 0.05) (Table 4).

All exposure groups and regimes yielded harmful consequences when compared to their respective controls (*P* < 0.05), as determined by the generalized biomarker response analysis through IBR calculation (Table 6, 7, and 8). The highest IBR value corresponds the most pronounced biological effect, but a decrease in IBR values can also indicate a negative impact of a toxicant in our experimental setting (Dr Simon Devin, *personal communication*). Therefore, both increases or decreases in IBR values compared to control groups signify harmful pollutant effects. These changes might be related to the contamination level or toxicant concentration.

**Table 6:** Summary of IBR values for subchronic exposure

	IBR.mean ± S.D	IBR.min	IBR.median	IBR.max
Control	4.87 ± 0.22	4.36	4.88	5.41
FLX_0.025TU_	8.56 ± 0.35^**ac**^	7.77	8.54	9.36
FLX_0.05TU_	3.12 ± 0.66^ac*^	1.54	3.18	4.74
Zn_0.05TU_	1.52 ± 0.51^**a**^	0.34	1.53	2.77
FLX_0.025TU_ + Zn_0.05TU_	0.53 ± 0.30^**ab**^	0.05	0.49	1.23
FLX_0.05TU_ + Zn_0.05TU_	8.11 ± 0.42^**abc**^	7.08	8.10	9.19

^**a**^Compared to the control (*P* < 0.05)

^**b**^Compared to the corresponding FLX_0.025TU_ or FLX_0.05TU_ group (*P* < 0.05)

^**c**^Compared to Zn_0.05TU_ group (*P* < 0.05)

^*^Compared to the lowest FLX concentration tested (*P* < 0.05)

Number of random samplings at each iteration = 5

**Table 7:** Summary of IBR values for acute exposure

	IBR.mean ± S.D	IBR.min	IBR.median	IBR.max
Control	8.77 ± 0.16	8.47	8.74	9.09
FLX_0.05TU_	5.72 ± 1.48^**c**^	2.87	5.65	8.71
FLX_0.1TU_	1.98 ± 0.86^a*^	0.41	1.89	3.76
Zn_0.1TU_	0.98 ± 0.65^**a**^	0.02	1.01	2.31
FLX_0.05TU_ + Zn_0.1TU_	1.10 ± 0.51^**ab**^	0.20	1.14	2.09
FLX_0.1TU_ + Zn_0.1TU_	5.44 ± 0.20^**bc**^	5.04	5.48	5.80

^**a**^Compared to the control (*P* < 0.05)

^**b**^Compared to the corresponding FLX_0.05TU_ or FLX_0.1TU_ group (*P* < 0.05)

^**c**^Compared to Zn_0.1TU_ group (*P* < 0.05)

^*^Compared to the lowest FLX concentration tested (*P* < 0.05)

Number of random samplings at each iteration = 5

**Table 8:** Summary of IBR values for WDL exposure

	IBR.mean ± S.D	IBR.min	IBR.median	IBR.max
Control	9.50 ± 0.34	8.83	9.49	10.14
FLX_0.05TU_	3.38 ± 0.72^**ac**^	2.00	3.40	4.87
FLX_0.1TU_	1.23 ± 0.45^ac*^	0.35	1.20	2.17
Zn_0.1TU_	6.27 ± 0.44^**a**^	5.30	6.25	7.22
FLX_0.05TU_ + Zn_0.1TU_	1.88 ± 0.50^**abc**^	0.80	1.84	2.85
FLX_0.1TU_ + Zn_0.1TU_	3.61 ± 0.72^**abc**^	2.16	3.57	5.19

^**a**^Compared to the control (*P* < 0.05)

^**b**^Compared to the corresponding FLX_0.05TU_ or FLX_0.1TU_ group (*P* < 0.05)

^**c**^Compared to Zn_0.1TU_ group (*P* < 0.05)

^*^Compared to the lowest FLX concentration tested (*P* < 0.05)

Number of random samplings at each iteration = 5

Following acute or subchronic in vivo exposures, damage from Zn^2+^ alone or in combination with FLX was enhanced (*P* < 0.05). On the contrary, the lowest effect was observed after WDL Zn^2+^ exposure. It is interesting to note that the highest FLX concentrations when combined with Zn^2+^ showed an improvement after acute in vivo and WDL exposures compared to control, single FLX, and/or Zn^2+^ groups. The IBR value decreased when FLX concentration was increased after in all exposure regimes (*P* < 0.05), indicating that the negative impacts produced in individual FLX groups were concentration-dependent.

**Fig. 1:**
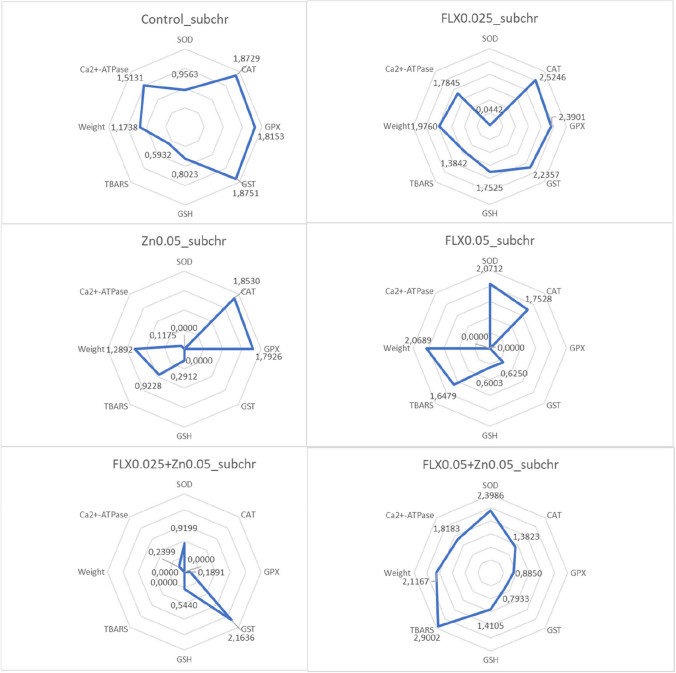
Radar graphic for integrated biomarker response index obtained in subchronic exposure. Each corner of the radar graph displays the standardized value for each biomarker

**Fig. 2:**
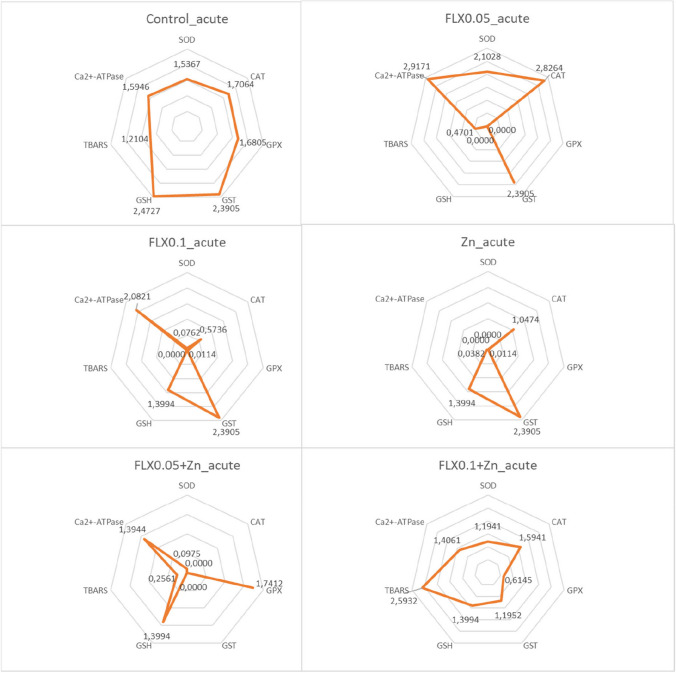
Radar graphic for integrated biomarker response index obtained in acute exposure. Each corner of the radar graph displays the standardized value for each biomarker

**Fig. 3:**
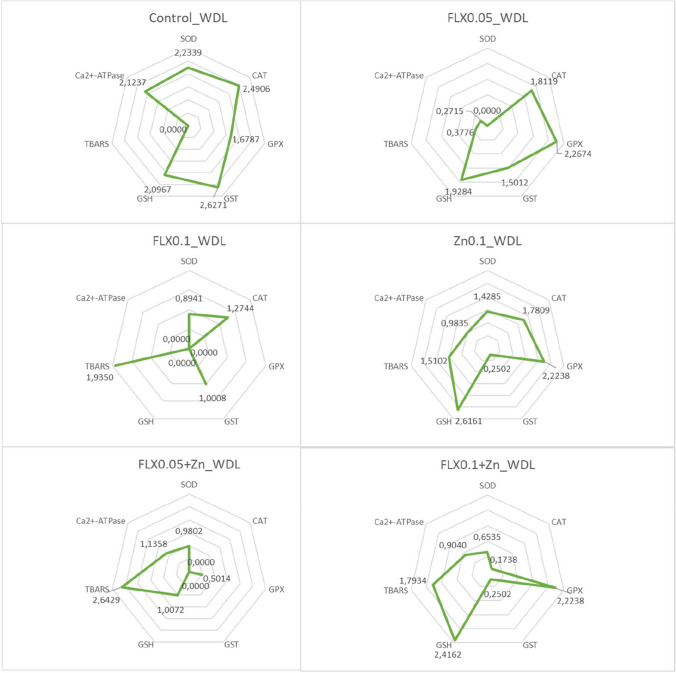
Radar graphic for integrated biomarker response index obtained in WDL exposure. Each corner of the radar graph displays the standardized value for each biomarker

In subchronic exposure regime, the most detrimental impact was observed in the FLX_0.025TU_ + Zn_0.05TU_ exposure group (Table 6), and CAT, GPX, GSH, TBARS, Ca^2+^-ATPase, and animal weight appear to be the factors that caused this effect (Fig. 1). In this group, all biomarkers, except GSH, exhibited a declining trend without statistical significance (Tables 1, 2, and 3). In acute exposure regime, Zn^2+^ demonstrated the greatest harmful consequences (Table 7), with its effects primarily driven by SOD, GPX, TBARS, and Ca^2+^-ATPase. A statistical analysis revealed that these biomarkers had already declined following acute Zn exposure (Table 4). The FLX_0.05TU_ + Zn_0.1TU_ group experienced decreased IBR values that were comparable to those of the Zn exposure, indicating that this group also experienced the effects of Zn. SOD, CAT, GST, GSH, and TBARS all had an impact on the IBR value in this group (Fig. 2). In WDL exposure regime, the most significant biological alteration is caused by FLX_0.1TU_ exposure (Table 8), influenced by all the parameters analysed (Fig. 3). A statistical analysis of all biomarkers in this group, except for GPX and GST enzyme activities, revealed a trend toward either a decreasing or an increasing pattern (Table 5).

## Discussion

In this study, the detrimental effects of both single and combined FLX and Zn were revealed in the non-target organism *D. magna*. This revelation was made possible through the sensitive responses of the antioxidant system and Ca^2+^-ATPase biomarkers along with IBR analyses. According to obtained results, it is suggested that the animals face substantial toxicity of Zn^2+^ and FLX, albeit not reaching lethal levels.

### Morphometric characteristics

Single FLX did not exert any influence on morphometric traits at the tested concentrations. However, FLX exacerbated the effects of single Zn^2+^ on animal weight. Muyssen et al. (2006) proposed that Zn-mediated inhibition of Ca^2+^ absorption led to restricted body Ca^2+^ content, subsequently causing a decline in food intake and, consequently, diminished energy reserves, which ultimately inhibited body growth. Reduced food intake may also be a cause of weight decrease. It was reported that a nonselective beta-blocker and serotonin antagonist propranolol reduced the feeding rate in mussel, *Mytilus galloprovincialis* (Solé et al. 2010). *D. magna* with a serotonin biosynthesis enzyme mutation displays lower growth rate (Rivetti et al. 2018). Therefore, decreased weight after combined FLX and Zn^2+^ exposure is thought to be caused by an imbalance in Ca^2+^ and serotonin levels; however, further mechanistic research should be conducted. Caudal spine length in FLX and Zn combination groups was attenuated compared to the single Zn^2+^ effect. In daphnids, this trait can be affected by chemical toxicity; for instance, lesser sulfoxaflor concentrations increased caudal spine length, while higher concentrations decreased it (Sevgiler and Atli 2022).

### Ca^2+^-ATPase activity

According to our results, FLX increased Ca^2+^-ATPase activity except in the lowest concentration tested in subchronic exposure and WDL exposures. Majeed et al. (2015) suggested that FLX increased cytosolic Ca^2+^ levels through the ER stores in the crayfish, *Procambarus clarkii*. Different mammalian cell types experienced an increase in the cytosolic Ca^2+^ content because of ER Ca^2+^ release driven on via FLX exposure. The mechanism may involve Ca^2+^ leakage from the translocon, which is located on the ER membrane. Because of Ca^2+^ release from the ER driven by the exposure to FLX, the mitochondrial membrane depolarizes and consequently oxygen consumption and ATP production will be reduced. Lower ATP levels then result in lower SERCA activity, and cytosolic Ca^2+^ levels rise (Charles et al. 2017). In our prior investigation, FLX_0.012TU_ and FLX_0.0015TU_ exposures in *D. magna* resulted in mitochondrial membrane depolarization after 96 h and 21 days (Över et al. 2020). Since Ca^2+^-ATPase balances cytosolic Ca^2+^ levels, induction of Ca^2+^-ATPase activity following FLX exposure is inevitable. In light of our previous study, the in vivo and WDL inhibition of Ca^2+^-ATPase activity should be concentration-dependent because of the unaltered activity after 48-h Zn_0.05TU_ exposure in *D. magna* (Sevgiler and Atli 2022). In the present study, this concentration also has no impact following subchronic exposure contrasting the inhibition after in vivo and WDL Zn_0.1TU_. Analyzing in vivo studies reveals a consistent trend of decreased Ca^2+^-ATPase activity in acute and chronic exposures to various metals and pesticides across different aquatic organisms (Rogers and Wood 2004; Atli and Canli 2007; Mahmut et al. 2022; Sevgiler and Atli 2022). It was concluded that Zn^2+^ may have inhibited Ca^2+^-ATPase activity by competing with Ca^2+^ for the Ca-selective channel, which caused an ion disruption (Heath 1995). Zn^2+^ has an inhibitory effect on the NMDA receptor complex in mammals, and this effect is attributed to its antidepressant function (Nowak 2015). The NMDA receptor complex in *D. pulex* is crucial for intracellular Ca^2+^ regulation and plays a pivotal role in the production of male offspring (Toyota et al. 2015). Zn^2+^ and FLX has complex relations on serotonin and nicotinic acetylcholine receptors (nAChRs), and the inhibitory potential of FLX on nAChRs is enhanced by Zn^2+^ co-exposure in mammals (for further details, see Nowak (2015). FLX ameliorated the inhibitory potential of Zn^2+^ on Ca^2+^-ATPase activity in *D. magna* after in vivo exposures in the current study. While complex interactions between Zn^2+^ and FLX on different receptor types, which affect the intracellular Ca^2+^ regulation, were found in mammals, the interaction mechanism of FLX and Zn^2+^ on cellular ion regulation in crustaceans should be a subject for further research. After the in vitro exposure to Zn^2+^, inhibition of Ca^2+^-ATPase activity was found in the rainbow trout gill (Hogstrand et al. 1996) and tilapia muscle (Atli and Canli 2013), which are in accordance with our data. It has been concluded that decreased heart rate in *D. magna* may through an inhibition of plasma Ca^2+^ and Na^+^ channels (if they exist) by SSRIs including FLX (Halliwushka 2016). However, we hypothesized that the suppression of Ca^2+^-ATPase activity following single or combination FLX exposure in the present investigation was due to the absence of complicated compensatory mechanisms in WDL experiments. This may also be partially a result of the toxicants' direct effects on the enzyme molecule and/or isolated structure in the absence of the whole-body physiological and biochemical processes that allow the organism to adapt to the toxicant effect (Atli and Canli 2013). To the best of our knowledge from the literature, this study offers a first and important source of data regarding the effect of single and binary exposure to FLX and metals on Ca^2+^-ATPase activity.

### Oxidative stress response

Variable response patterns were observed due to exposure duration (acute or subchronic) and treatment condition (in vivo or WDL), single and binary exposure, concentration levels, and toxicant type differences such as reverse effect or non-responsive situations for some cases.

It is suggested that the SOD and CAT inhibitory effects of all toxicants are duration specific. Inhibited SOD activity was also noticed in *D. magna* in our prior study following acute in vivo and WDL Zn_0.05TU_ exposure (Sevgiler and Atli 2022). Exaggerated Zn^2+^ levels can inhibit certain glycolytic enzymes and components of the electron transport chain, leading to the production of ROS (Lee 2018). In turn, more H_2_O_2_ can reduce SOD activity (Ma et al. 2017). Inhibitory effects of FLX on SOD activity have been documented in the literature in a variety of aquatic invertebrates, including mussels, clams, and daphnids (Gonzalez-Rey and Bebianno 2013; Chen et al. 2015; Ding et al. 2017; Magni et al. 2017). In mammals, FLX induced mitochondrial electron leakage and affected the mitochondrial redox parameters to induce ROS formation (de Oliveira 2016). Oxidative modification can cause the loss of function due to their protein nature of antioxidant enzymes, and their gene expression may be induced as a compensation mechanism. SSRIs have been shown to increase the synthesis of antioxidant enzymes. For example, 500 ng/L FLX exposure increased SOD, CAT, and GPX mRNA expression levels in zebrafish larvae while GST expression did not change (Parolini et al. 2019). The decrease in CAT activity should mostly be attributed to the reduced SOD activity brought on by substrate limitation. Mitochondrial effects of FLX was also reported in *D. magna*, as evidenced by the loss of mitochondrial membrane polarization (MMP) and higher TBARS levels that signal the ROS production in our previous study (Över et al. 2020). Although the precise mechanism of ROS formation in FLX-exposed non-target aquatic invertebrates is unknown, it may be connected to uncompensated cytosolic Ca^2+^ levels and loss of MMP, while Ca^2+^-ATPase, at least in acute exposure, did not support this hypothesis. Declined SOD and CAT activities could be attributed to the loss of ability to compensate for the effect of the enhanced amount of ROS. Our data demonstrated a positive correlation (*r*^2^ = 0.96, *P* < 0.05) between the loss in GSH and SOD activity following subchronic Zn_0.05TU_ exposure. Due to acute Zn combination groups consuming all of their GSH, such a correlation could not be assessed. In our previous research, the sulfoxaflor and Zn_0.05TU_ effect on *D. magna* antioxidant system also indicated the correlations of GSH with SOD and CAT upon acute and subchronic durations. This could highlight the crucial function that GSH levels play in the antioxidant system's response. The GSH function may also be important after subchronic FLX_0.025TU_ exposure. In this group, SOD activity remained unaffected while CAT activity decreased and GSH levels increased.

Besides, increases in GSH levels after subchronic FLX_0.025TU_ and WDL FLX_0.1TU_ could be associated with increased tolerance to cope with oxidative stress. Byeon et al. (2020) reported GSH alterations in the marine rotifer *Brachionus koreanus* in response to FLX and sertraline, two antidepressants that caused both augmentation and reduction, respectively. Following acute (4-h) and chronic (42 days) 200 µg/L FLX exposure, the GSH-GSSG system in the liver of fish *Pseudorasbora parva* was likewise identified as being most impacted among all antioxidants studied (Chen et al. 2018). GSH reduction could indicate its consumption in order to decrease the cytotoxic effects and may be brought on by the loss of adaptive mechanisms. It is therefore regarded as a sensitive indicator of oxidative damage in aquatic organisms (Atli et al. 2020).

Following exposure to FLX_0.012TU_ and FLX_0.0015TU_ for 96-h and 21 days in *D. magna* in vivo, the GPX activity was increased (Över et al. 2020). Similarly, Orozco-Hernández et al. (2022) found that 96-h exposure increased lipid peroxidation levels, and SOD, CAT, and GPX activities in *Danio rerio* embryos at concentrations between 15 and 40 ng/L. Therefore, while evaluating the effects of FLX alone on GPX activity, it could be required to take the concentration-dependence into account. However, the antioxidant system has a complicated structure, and before drawing meaningful conclusions, various elements that are impractical to examine all at once must be considered. For instance, GPX and CAT activities compete in their substrate, which is H_2_O_2_, and GPX activity depends on its substrate, which is GSH. Nevertheless, a negative correlation (*r*^2^ =  − 1.00, *P* < 0.05) was found between CAT and GPX activity after acute FLX_0.1TU_ exposure. Our earlier investigation found that *D. magna* had increased GPX and decreased SOD activities upon subchronic Zn_0.05TU_ exposure (Sevgiler and Atli 2022), while GPX activity was found to be unaffected in the current research. Therefore, we propose that differentiation in the outcomes is typical and that compensation mechanisms divaricate to counteract ROS effects. In both acute and WDL tests, exposure to Zn_0.1TU_ reduced GPX activity, whereas combined exposure to FLX had no impact on the decreased activity after Zn^2+^ exposure. A positive correlation in acute Zn_0.1TU_ group was accompanied by decreased SOD and GPX activity (*r*^2^ = 1.00, *P* < 0.05) in the present study. After acute or WDL exposures, decreased SOD and GPX activities in the Zn^2+^ alone or Zn + FLX combination groups may have indicated ROS-inducing potentials of FLX and Zn, which were also associated by greater lipid peroxidation levels in these groups.

In contrast to the other antioxidant system parameters analysed, GST activity was found to be less susceptible to in vivo and WDL FLX and Zn^2+^ exposures. However, subchronic exposures to Zn_0.05TU_ and FLX_0.05TU_ + Zn_0.05TU_ caused a reduction in its activity. This decrease may be linked to GSH loss, which is a sign of consumption. According to Chen et al. (2018), GST activity was induced in the gills of the fish *P. parva* after 4-h of exposure to 200 µg/L FLX, as opposed to being inhibited in the liver and gills after 42 days of exposure. This decline was consistent with the antioxidant capacity being exceeded during long-term FLX exposure, which was also linked to an increase in the levels of lipid peroxidation in these tissues (Chen et al. 2018). A possible connection between the increased TBARS levels and the decline in GST activity in *D. magna* following subchronic exposure to Zn_0.05TU_ and FLX_0.05TU_ + Zn_0.05TU_ is plausible in the current investigation. Similar to our findings, the GST activity in common goby *Pomatoschistus microps* was unaffected by short-term (96-h) FLX concentrations of 0.1, 0.5, 10, and 100 µg/L. It was concluded that these FLX concentrations did not generate overt oxidative stress in *P. microps*, which was also in association with lack of lipid peroxidation changes. Therefore, GST should not be considered a reliable biomarker for FLX’s oxidative effects.

Increased ROS levels after subchronic FLX_0.05TU_ + Zn_0.05TU_ exposure, which drastically decreased the GSH levels in *D. magna*, may have contributed to the rise in TBARS levels. WDL experiments also revealed a decline in GSH levels along with suppressed SOD activity, particularly in the Zn^2+^ alone or combination groups. Actually, FLX and its ultimate target serotonin operate as in silico hydroxyl and peroxyl radical scavengers (Muraro et al. 2019). This suggests that FLX’s antioxidant capability may be important pharmacological mechanism in its mode of action (Novío et al. 2011). In contrast, FLX has led to adverse effects such as nose contraction and paralysis in the nematode *Caenorhabditis elegans* under in vivo conditions and at high concentrations, independently of serotonin (Ranganathan et al. 2001). Thus, serotonin accumulation may not be an important mediator of the prooxidative effects of FLX. High ROS levels in the liver of crucian carp *Carassius auratus* can be inferred with high lipid peroxidation levels and insufficient antioxidant capacity to defend against the oxidative stress caused by FLX at its environmental or higher concentrations (Ding et al. 2016). It was stated that loss in SOD activity was likely due to the elevation in SOD-consuming superoxide anion radicals produced by FLX effect; therefore, the elevated TBARS levels become apparent (Gonzalez-Rey and Bebianno 2013; Chen et al. 2015; Ding et al. 2016). The current data also provided support for FLX-induced lipid peroxidation in aquatic invertebrates, demonstrated by exposure to 5 and 50 µg/L FLX for 30 days in adult Asian clam *Corbicula fluminea* (Chen et al. 2015), and 5.4 µg/L for 14 days in juvenile oysters *Crassostrea gigas* (Di Poi et al. 2016). Despite the unchanged TBARS content in *M. galloprovincialis* after 7 days of 75 ng/L FLX exposure, a significant increase was recorded after 15 days of exposure (Gonzalez-Rey and Bebianno 2013). Consistent with these observations, FLX at lower concentrations increased lipid peroxidation levels over longer durations. Our earlier findings showed that 96-h and 21 days of FLX_0.012TU_ and FLX_0.0015TU_ exposures, respectively, increased the levels of lipid peroxidation in *D. magna* (Över et al. 2020). This highlights the importance of accounting for the exposure duration when evaluating the impact of FLX, either alone or in conjunction with Zn, on lipid peroxidation levels.

The current investigation discovered complex response patterns in individual biomarkers following exposure to various combinations of FLX, Zn, or their mixtures during WDL and subchronic or acute in vivo exposures. The multi-biomarker technique can be used to assess the effects of a single toxicant or toxicant combinations on numerous biological systems while incorporating the overall trend of “effects on biomarkers.” In this regard, IBR analysis leads us to the conclusion that Zn^2+^ at the tested concentrations was fairly effective on *D. magna* in subchronic and acute in vivo experiments; nevertheless, WDL Zn^2+^ exposure had a lower impact compared to these exposure durations. This result suggests that Zn needs intact biological response mechanism to reveal its toxicity. Due to the lowest IBR values, the observed effect of Zn^2+^ on *D. magna* is congruent with its harmonized classification codes (H400, H410) even at low environmental concentrations (0.05TU and 0.1TU for subchronic and acute periods, respectively). The H400 and H410 indicates that the chemical is extremely harmful to aquatic life in both short- and long-term exposures under the GHS (Global Harmonized System of Classification and Labelling of Chemicals, United Nations) classification. The harmonized classification codes H400 and H410 are also assigned to FLX. Based on current data and previous researches in invertebrates (De Castro-Català et al. 2017; Över et al. 2020), as well as findings from aquatic vertebrates such as zebrafish (Orozco-Hernández et al. 2023), FLX poses an extreme hazard to aquatic organisms even at very low environmental concentrations following both short- and long-term exposures. In fact, FLX_0.025TU_ (the study’s lowest concentration) had the greatest harmful impact on overall health status of *D. magna* over the course of seven days, both alone and in combination with Zn^2+^ according to IBR analysis. Environmental specialists should pay particular attention to the low FLX concentrations because these levels are the most likely to be detected in various aquatic systems. The counter impact of Zn^2+^ and FLX on serotonin absorption mechanisms, as was reported in mammalian studies (García-Colunga et al. 2005), can be linked to the observed improvements in IBR values following acute in vivo and WDL exposures. The diverse directions of FLX and Zn^2+^ in the IBR values of the single and combined groups after the subchronic period may also serve as evidence for the different impacts of Zn^2+^ and FLX. The involvement of different response pathways, which warrants further investigation, may also contribute to the contradictory directions observed in IBR results after FLX exposure. At higher concentrations (0.22–0.44 mM), FLX induced effects in a nematode *Caenorhabditis elegans* that are independent of SERT and serotonin, such as nose contraction and paralysis (Ranganathan et al. 2001). This suggests that FLX may influence various response pathways depending on its concentration, rather than solely acting on serotonergic pathways as was already mentioned previously. Even though FLX is considered safe and useful for a variety of problems in humans, it should be seriously considered because both its single and combined effects resulted in significant changes in biomarker levels leading to failures in the antioxidant system and Ca^2+^-ATPase functions, two crucial processes for daphnid species.

In conclusion, the environmental risks associated with FLX and Zn^2+^, two substances that are similar in their ability to act as an antidepressant in humans, overlap. The provided data has shown the potential pro-oxidative and anti-Ca^2+^-ATPase effects of FLX and Zn^2+^ on *D. magna*, impacting both Ca^2+^-ATPase activity and the antioxidant system across all exposure durations and patterns. IBR analysis revealed that Zn^2+^ was reasonably effective on the health of *D. magna*, particularly following in vivo exposures, and FLX toxicity showed a concentration-dependent increase, but was reversed by the combined exposure. The observed adverse effects in Daphnia may also have harmful impact on ecosystem levels due to ecological significance of non-target *D. magna*, holds environmental value for higher trophic levels due to its ecological significance. Pharmaceutical exposure, such as FLX, should therefore be considered even at low environmental concentrations (ng to µg/L). Therefore, it is crucial to assess their toxicity and interactions with other aquatic toxins, as these substances may disrupt vital biological systems in aquatic organisms.

### Supplementary Information

Below is the link to the electronic supplementary material.

Supplementary file1 (DOCX 31 KB)

## Data Availability

Not applicable.
