# Supplementary material for: Binary effects of fluoxetine and zinc on the biomarker responses of the non-target model organism Daphnia magna
Source: Environ Sci Pollut Res Int. 2024 Mar 25;31(19):27988–8006. doi: 10.1007/s11356-024-32846-5 (PMC11058962; doi:10.1007/s11356-024-32846-5)
Supplement: Supplementary file 1 — Supplementary file1 (DOCX 31 KB) [file 11356_2024_32846_MOESM1_ESM.docx]

**Table S1.** ANOVA table obtained from subchronic exposure statistical analysis.

| **ANOVA Table for Subchronic Exposure** | | | | | | |
| --- | --- | --- | --- | --- | --- | --- |
|  | | Sum of Squares | df | Mean Square | F | Sig. |
| **CarLen** | Between Groups | 295.100 | 5 | 59.020 | .642 | .670 |
|  | Within Groups | 2205.600 | 24 | 91.900 |  |  |
|  | Total | 2500.700 | 29 |  |  |  |
| **CarWid** | Between Groups | 769.067 | 5 | 153.813 | 1.639 | .188 |
|  | Within Groups | 2252.400 | 24 | 93.850 |  |  |
|  | Total | 3021.467 | 29 |  |  |  |
| **SpiLen** | Between Groups | 506.667 | 5 | 101.333 | 2.834 | .038 |
|  | Within Groups | 858.000 | 24 | 35.750 |  |  |
|  | Total | 1364.667 | 29 |  |  |  |
| **Weigth** | Between Groups | 1.399 | 5 | .280 | 11.479 | .000 |
|  | Within Groups | .585 | 24 | .024 |  |  |
|  | Total | 1.984 | 29 |  |  |  |
| **CaATPase** | Between Groups | 18.893 | 5 | 3.779 | 4.002 | .014 |
|  | Within Groups | 16.051 | 17 | .944 |  |  |
|  | Total | 34.945 | 22 |  |  |  |
| **Protein** | Between Groups | 4.387 | 5 | .877 | .866 | .518 |
|  | Within Groups | 24.312 | 24 | 1.013 |  |  |
|  | Total | 28.699 | 29 |  |  |  |
| **CAT** | Between Groups | 258.139 | 5 | 51.628 | 2.981 | .033 |
|  | Within Groups | 381.008 | 22 | 17.319 |  |  |
|  | Total | 639.147 | 27 |  |  |  |
| **TBARS** | Between Groups | .092 | 5 | .018 | 2.911 | .034 |
|  | Within Groups | .151 | 24 | .006 |  |  |
|  | Total | .243 | 29 |  |  |  |
| **SOD** | Between Groups | 14.808 | 5 | 2.962 | 2.866 | .036 |
|  | Within Groups | 24.799 | 24 | 1.033 |  |  |
|  | Total | 39.607 | 29 |  |  |  |
| **GPX** | Between Groups | .000 | 5 | .000 | 3.872 | .012 |
|  | Within Groups | .000 | 21 | .000 |  |  |
|  | Total | .000 | 26 |  |  |  |
| **GST** | Between Groups | .000 | 5 | .000 | 4.006 | .009 |
|  | Within Groups | .000 | 24 | .000 |  |  |
|  | Total | .000 | 29 |  |  |  |
| **GSH** | Between Groups | 10.587 | 5 | 2.117 | 15.164 | .000 |
|  | Within Groups | 2.653 | 19 | .140 |  |  |
|  | Total | 13.240 | 24 |  |  |  |

**Table S2.** ANOVA table obtained from acute exposure statistical analysis.

| **ANOVA Table for Acute Exposure** | | | | | | |
| --- | --- | --- | --- | --- | --- | --- |
|  | | Sum of Squares | df | Mean Square | F | Sig. |
| **CAT** | Between Groups | 128.792 | 5 | 25.758 | 4.340 | .007 |
|  | Within Groups | 124.647 | 21 | 5.936 |  |  |
|  | Total | 253.439 | 26 |  |  |  |
| **Protein** | Between Groups | 7.141 | 5 | 1.428 | 6.243 | .001 |
|  | Within Groups | 5.490 | 24 | .229 |  |  |
|  | Total | 12.631 | 29 |  |  |  |
| **TBARS** | Between Groups | .252 | 5 | .050 | 2.818 | .041 |
|  | Within Groups | .393 | 22 | .018 |  |  |
|  | Total | .645 | 27 |  |  |  |
| **SOD** | Between Groups | 120.721 | 5 | 24.144 | 4.955 | .004 |
|  | Within Groups | 102.334 | 21 | 4.873 |  |  |
|  | Total | 223.055 | 26 |  |  |  |
| **GPX** | Between Groups | .001 | 5 | .000 | 30.302 | .000 |
|  | Within Groups | .000 | 17 | .000 |  |  |
|  | Total | .001 | 22 |  |  |  |
| **GST** | Between Groups | .000 | 5 | .000 | .131 | .984 |
|  | Within Groups | .001 | 24 | .000 |  |  |
|  | Total | .001 | 29 |  |  |  |
| **GSH** | Between Groups | .527 | 5 | .105 | 48.067 | .000 |
|  | Within Groups | .046 | 21 | .002 |  |  |
|  | Total | .574 | 26 |  |  |  |
| **CaATPase** | Between Groups | 232.567 | 5 | 46.513 | 5.847 | .002 |
|  | Within Groups | 143.188 | 18 | 7.955 |  |  |
|  | Total | 375.755 | 23 |  |  |  |

**Table S3.** ANOVA table obtained from WDL exposure statistical analysis.

| **ANOVA Table for WDL Exposure** | | | | | | |
| --- | --- | --- | --- | --- | --- | --- |
|  | | Sum of Squares | df | Mean Square | F | Sig. |
| **CAT** | Between Groups | 45.861 | 5 | 9.172 | 4.912 | .003 |
|  | Within Groups | 44.819 | 24 | 1.867 |  |  |
|  | Total | 90.680 | 29 |  |  |  |
| **SOD** | Between Groups | 15.588 | 5 | 3.118 | 11.092 | .000 |
|  | Within Groups | 6.746 | 24 | .281 |  |  |
|  | Total | 22.334 | 29 |  |  |  |
| **GST** | Between Groups | .000 | 5 | .000 | 1.245 | .320 |
|  | Within Groups | .000 | 24 | .000 |  |  |
|  | Total | .000 | 29 |  |  |  |
| **TBARS** | Between Groups | .005 | 5 | .001 | 3.526 | .024 |
|  | Within Groups | .005 | 16 | .000 |  |  |
|  | Total | .010 | 21 |  |  |  |

**Table S4.** Kruskal-Wallis table obtained from WDL exposure statistical analysis.

| **Kruskal-Wallis Table for WDL Exposure** | | | |
| --- | --- | --- | --- |
| **Ranks** | | | |
|  | group | *N* | Mean Rank |
| **Ca-ATPase** | 1 | 4 | 22.50 |
|  | 2 | 4 | 15.00 |
|  | 3 | 4 | 17.50 |
|  | 4 | 4 | 7.00 |
|  | 5 | 4 | 5.00 |
|  | 6 | 4 | 8.00 |
|  | Total | 24 |  |
|  | df | 5 | |
| **GPX** | 1 | 5 | 18.80 |
|  | 2 | 5 | 22.30 |
|  | 3 | 5 | 24.90 |
|  | 4 | 5 | 7.50 |
|  | 5 | 4 | 7.50 |
|  | 6 | 5 | 7.50 |
|  | Total | 29 |  |
|  | df | 5 | |
| **GSH** | 1 | 5 | 17.60 |
|  | 2 | 3 | 17.00 |
|  | 3 | 5 | 23.80 |
|  | 4 | 4 | 4.50 |
|  | 5 | 4 | 7.00 |
|  | 6 | 5 | 9.40 |
|  | Total | 26 |  |
|  | df | 5 | |
